# Effects of Elective Percutaneous Coronary Intervention on Subtle Left Ventricular Systolic Dysfunction in Patients With Stable Coronary Artery Disease as Assessed by Global Longitudinal Strain Imaging

**DOI:** 10.7759/cureus.75133

**Published:** 2024-12-05

**Authors:** Swargam Venu, Ramnath Venkitasubramonia Iyer, Prabath Kumar Dash

**Affiliations:** 1 Cardiology, Dr. Pinnamaneni Siddhartha Institute of Medical Sciences and Research Foundation, Vijayawada, IND; 2 Cardiology, Sri Sathya Sai Institute of Higher Medical Sciences, Bengaluru, IND

**Keywords:** cardiovascular diseases, coronary artery disease, global longitudinal strain, left ventricular systolic dysfunction, percutaneous coronary intervention

## Abstract

Aim

The study aimed to detect subtle left ventricular (LV) systolic dysfunction, reflected by abnormal global longitudinal strain (GLS), in patients with stable coronary artery disease (CAD) undergoing percutaneous coronary intervention (PCI) and to evaluate any improvement in GLS at 24 hours and six months post-PCI.

Methods

A total of 94 patients with stable CAD scheduled for elective PCI at our hospital were evaluated using conventional 2D echocardiography and GLS prior to the procedure. Follow-up assessments were conducted at 24 hours and six months post-PCI.

Results

Our study revealed evidence of subclinical LV dysfunction in the form of reduced baseline GLS (-16.72 ± 1.98) despite a normal ejection fraction (59.21 ± 2%). Baseline GLS showed a significant correlation with the severity of CAD, declining progressively with an increasing number of stenotic coronary vessels. Notably, there was significant improvement in subclinical LV dysfunction following PCI, as evidenced by enhanced GLS values at the six-month follow-up (-21.87 ± 1.70; p < 0.001). Among patients stratified into single-vessel disease (SVD, 63.8%), double-vessel disease (29.8%), and triple-vessel disease (3VD, 6.4%), GLS improved significantly at both 24 hours and six months post-PCI compared to baseline.

Conclusions

Patients with stable CAD, normal LVEF, and no regional wall motion abnormalities on 2D echocardiography were found to exhibit subtle LV dysfunction as detected by GLS. GLS parameters showed significant improvement following successful PCI. The degree of GLS impairment progressively worsened with the severity of CAD, increasing from SVD to 3VD.

## Introduction

Over the years, cardiovascular diseases (CVDs) have remained the leading cause of death worldwide. Currently, CVD is responsible for approximately 17.3 million deaths annually, with projections suggesting this number will rise to 26.3 million by 2030 [1]. Lifestyle factors, including the increasing prevalence of diabetes, obesity, and hypertension, contribute to a higher risk of CVD-related deaths. While CVD is a global issue, in India, early-onset diabetes and smoking among young males are driving the early onset of coronary artery disease (CAD).

The diagnosis of stable CAD in patients is based on specific criteria, including the presence of angina, a positive exercise test indicating myocardial ischemia, and the confirmation of coronary artery stenosis through coronary angiography (CAG). In stable CAD patients, ischemia can lead to a progressive decline in systolic function [2]. Left ventricular (LV) systolic function plays a crucial role in predicting long-term survival in CAD patients [3], which has led to the growing popularity of non-invasive methods for evaluating cardiac function.

Echocardiography is essential in assessing both global and segmental LV systolic function in CAD patients. However, it is important to recognize that conventional echocardiographic parameters, such as LV ejection fraction (LVEF) and wall motion score index (WMSI), which depend on visual assessment of ventricular wall motion, may appear normal in a significant percentage of cases (ranging from 25% to 76%) [4]. Transthoracic echocardiography may be nondiagnostic in up to half of the patients presenting with suspected CAD. Therefore, evaluating regional and global subclinical LV dysfunction can be a valuable strategy for identifying myocardial regions with impaired coronary artery flow and reduced myocardial perfusion.

A novel imaging technique, 2D speckle-tracking echocardiography (2D-STE), has gained prominence for its ability to assess regional myocardial function in detail, enabling the detection of subtle global and segmental LV dysfunction. Notably, 2D-STE reduces interobserver variability and offers excellent accuracy, making it a reliable diagnostic tool [5]. The assessment of global longitudinal strain (GLS) using 2D-STE is highly reproducible compared to other parameters, such as LVEF or WMSI. This technique holds great promise for detecting subtle LV dysfunction caused by ischemia. By evaluating longitudinal myocardial deformation, GLS offers enhanced sensitivity, enabling more accurate diagnosis and management of patients [6-8]. GLS is particularly helpful in identifying subclinical LV dysfunction in ischemic and non-ischemic conditions, such as diabetes, which may later progress to overt LV dysfunction. Compared to conventional methods, GLS minimizes interobserver variability and can indicate procedural success when performed post-percutaneous coronary intervention (PCI). If GLS improves from baseline, it signals that PCI has positively impacted the subclinical dysfunction, which is associated with a better prognosis.

Over the past three decades, the use of PCI to treat ischemic CAD has increased dramatically [9]. Assessing the potential benefits of PCI in these patients requires evaluating baseline and post-PCI LV function [10,11]. Our study aimed to identify LV systolic dysfunction in the form of deranged GLS in patients with stable CAD and to assess any improvement in GLS at 24 hours and six months post-PCI.

## Materials and methods

Patients and study design

This was a prospective, observational, single-center study conducted at Sri Sathya Sai Institute of Higher Medical Sciences in Bengaluru, India, including patients with stable CAD who underwent PCI between July 2018 and December 2019. Patients who met the study eligibility criteria were considered for enrollment. All participants underwent conventional 2D echocardiography and 2D-STE for GLS assessment before PCI, as well as 24 hours and six months post-procedure.

**Figure 1:**
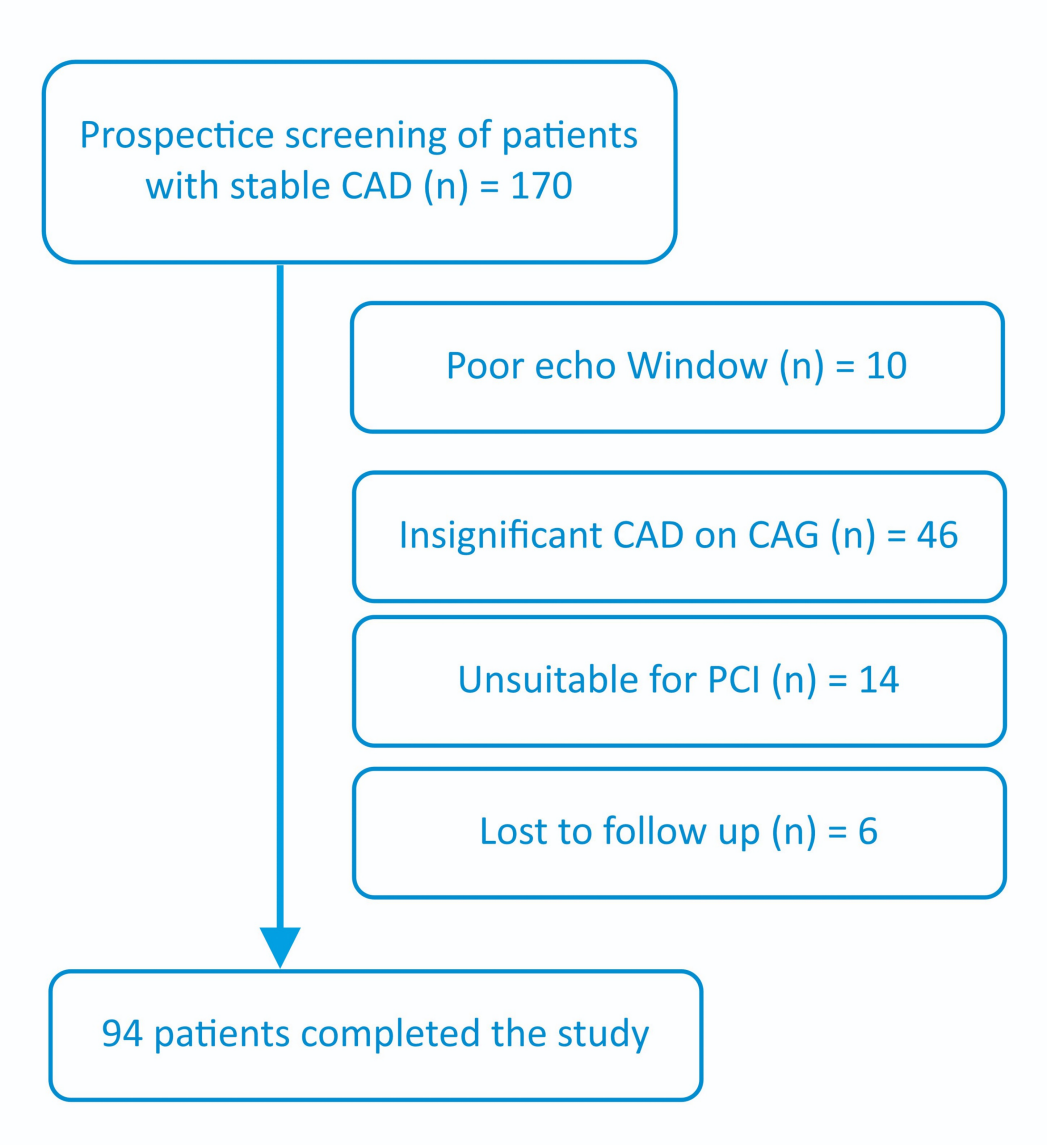
Study flow chart CAD: coronary artery disease; CAG: coronary angiography; PCI: percutaneous coronary intervention

Written informed consent was obtained from all patients in their native language at the time of enrollment. The study received approval from the hospital’s Ethics Committee prior to its initiation. A total of 170 subjects were screened for the study, and 94 subjects who met the inclusion criteria and completed all study-related procedures were included in the final analysis, as shown in Figure 1.

Inclusion criteria

Patients aged over 18 years, diagnosed with stable CAD based on symptomatology, who were scheduled for elective CAG followed by PCI, and who provided written informed consent were included. Stable CAD patients in this study had no acute coronary syndrome or chest pain despite optimal medical therapy (OMT).

Exclusion criteria

Exclusion criteria included patients with acute non-ST-segment elevation myocardial infarction (MI), acute ST-segment elevation MI, echocardiographic regional wall motion abnormality (RWMA; WMSI > 1), LV dysfunction, arrhythmias, a history of previous MI or coronary artery bypass grafting (CABG), left bundle branch block, severe valvular disease as per American Society of Echocardiography criteria, use of drugs affecting cardiac function (e.g., cytotoxic drugs), and patients with poor acoustic windows unsuitable for STE. The exclusion of patients with poor acoustic windows was vital for obtaining high-quality echocardiographic images required for accurate GLS assessment.

Echocardiographic procedure

All echocardiograms were performed using a Vivid E9 XD Clear echo machine (GE Vingmed, Horton, Norway) equipped with a 2.5 MHz transducer and version BT13 software. Conventional 2D echocardiography was conducted, and 2D-STE was analyzed offline using commercially available EchoPACS software (GE Vingmed) by a single blinded investigator.

Follow-up

Echocardiographic analysis was performed 24 hours after PCI, and again six months after discharge. All parameters were reassessed at these intervals. No adverse events, complications, or medication changes occurred during the follow-up period that could influence GLS. There were no dropouts.

Statistical analysis

The data were entered into Microsoft Excel spreadsheets (Microsoft Corporation, Redmond, WA, USA) and analyzed using IBM SPSS Statistics for Windows, Version 22.0 (Released 2013; IBM Corp., Armonk, NY, USA). Descriptive statistics were presented as frequency and percentage for categorical variables, and mean, median, standard deviation, and quartiles for continuous variables. Variations in GLS scores over different periods were compared using repeated-measures ANOVA, followed by the Bonferroni post hoc test. GLS scores between independent groups were compared using one-way ANOVA, followed by Tukey post hoc test. A p-value of <0.05 was considered statistically significant.

## Results

The average age of the study population was 51.85 ± 7.2 years, with 73.4% (n = 69) being male. Among the study participants, dyslipidemia was the most prevalent risk factor, present in 62.8% (n = 59) of patients, followed by hypertension, which was observed in 55.3% (n = 52) of patients (Table 1). Risk factors were self-reported by patients, and in some cases, they were clinician-diagnosed. Smoking included both active smokers and those with a history of smoking.

**Table 1:** Baseline demographic characteristics of the study population The data are represented as n (%) and mean ± SD.

Variables	Patients (n = 94)
Age (mean ± SD, years)	51.85 ± 7.21
Male, n (%)	69 (73.4%)
BMI (mean ± SD, kg/m^2^)	25.11 ± 3.14
Heart rate (mean ± SD, /min)	79.02 ± 5.81
Systolic blood pressure (mean ± SD, mmHg)	130.79 ± 13.14
Diastolic blood pressure (mean ± SD, mmHg)	81.96 ± 7.69
Hemoglobin (mean ± SD, gm/dl)	13.43 ± 1.57
Creatinine (mean ± SD, mg/dl)	0.86 ± 0.15
Hypertension, n (%)	52 (55.3%)
Diabetes, n (%)	35 (37.2%)
Dyslipidemia, n (%)	59 (62.8%)
Smoking, n (%)	27 (28.7%)

Table 2 presents the baseline echocardiographic parameters, demonstrating that the GLS in the study population before PCI was deranged, with a value of -16.72 ± 1.98, despite a normal EF (59.21 ± 2%) and normal LV internal dimensions (LV end-diastolic diameter: 41.87 ± 1.57 mm; LV end-systolic dimension: 25.63 ± 0.95 mm). This GLS value is lower than the reference normal threshold of <17.75. GLS proves to be a more sensitive measure, as it detects abnormalities even when the EF is normal. Pre-PCI GLS values were consistent across all subgroups.

**Table 2:** Baseline echocardiographic characteristics of the study population The data are represented as n (%) and mean ± SD. 2D echo EF: two-dimensional echocardiography-ejection fraction; E/A: early to atrial filling velocity ratio; E/e': early mitral inflow velocity to early diastolic mitral annulus velocity ratio; GLS: global longitudinal strain; LVEDD: left ventricular end-diastolic diameter; LVESD: left ventricle end-systolic dimension; PCI: percutaneous coronary intervention

Variables (mean ± SD)	Patients (n = 94)
GLS before PCI	-16.72 ± 1.98
2D echo EF (%)	59.21 ± 2.00
LVEDD (mm)	41.87 ± 1.57
LVESD (mm)	25.63 ± 0.95
E/A	1.06 ± 0.40
Deceleration time	188.7 ± 9.93
E/e'	10.83 ± 1.62

**Figure 2:**
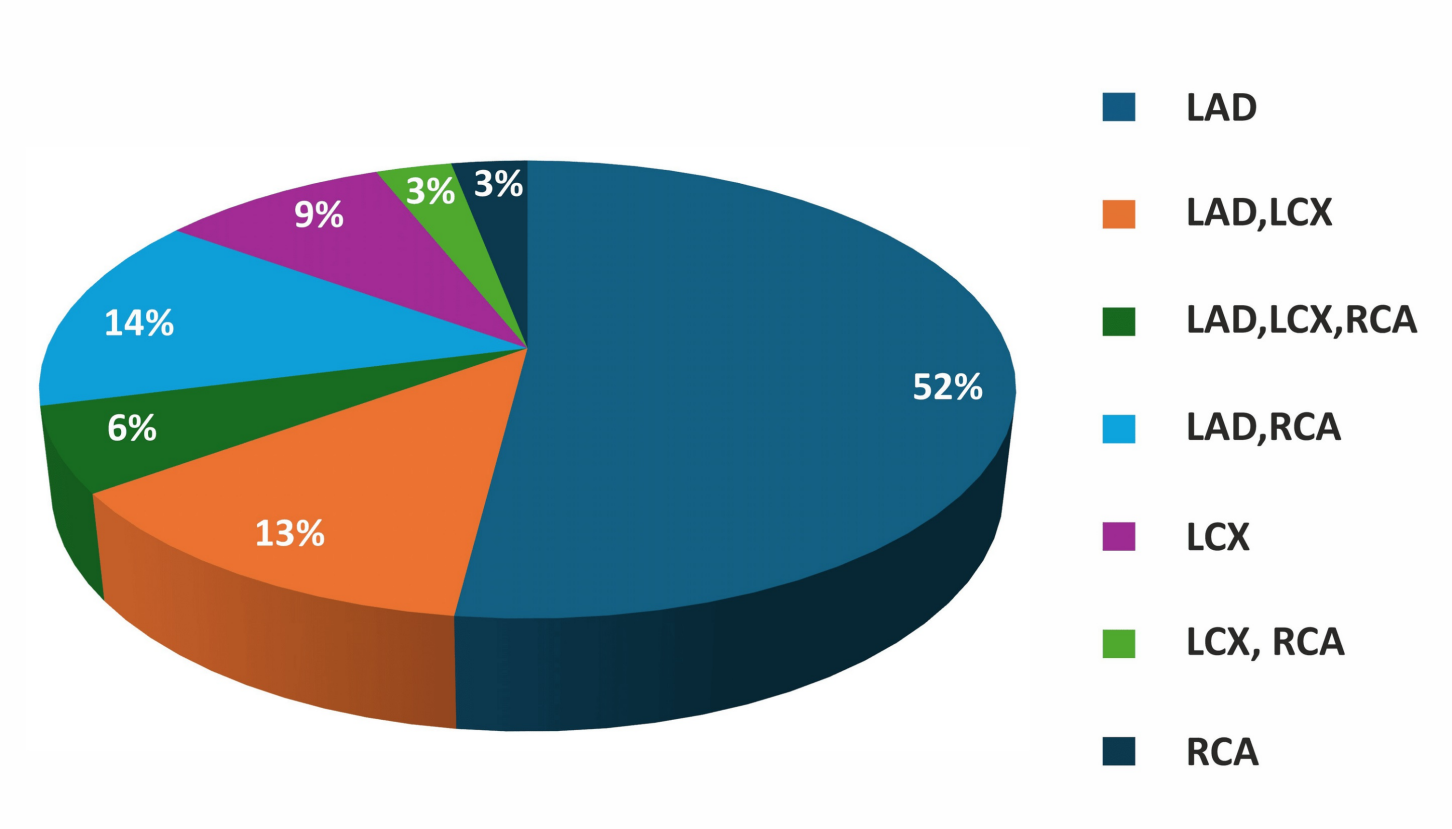
Comparison of LVEF (%) measured by the modified Simpson's method at 24 hours and six months post-PCI LAD: left anterior descending artery; LCX: left circumflex artery; LVEF: left ventricular ejection fraction; PCI: percutaneous coronary intervention; RCA: right coronary artery

A study of EF in the study population at different time intervals before and after intervention revealed that LVEF remained within normal limits at various time points. Specifically, LVEF was 59.21 ± 2% before PCI, 59.83 ± 1.79% at 24 hours post-PCI, and 60.69 ± 1.70% at six months post-PCI (Figure 2).

**Figure 3:**
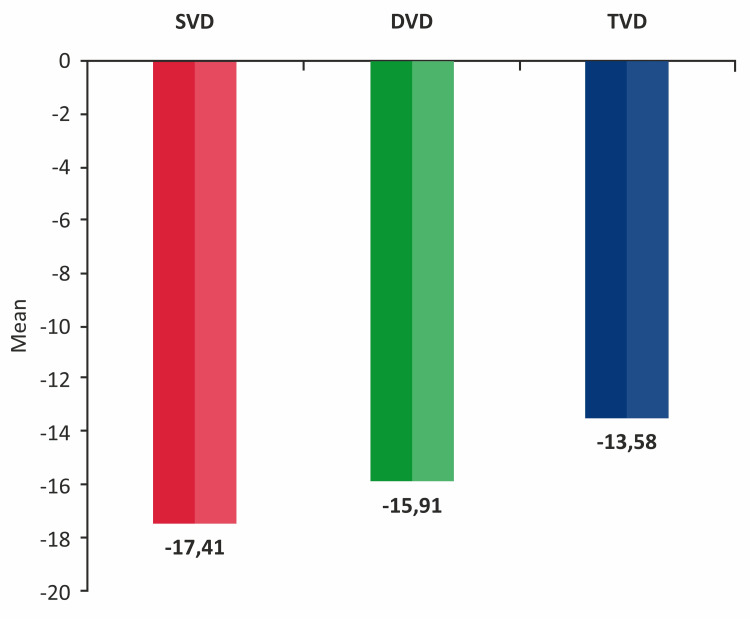
Distribution of study participants according to coronary arteries with stenosis DVD: double-vessel disease; SVD: single-vessel disease; TVD: triple-vessel disease

Patients with single-vessel disease (SVD) constituted the majority of the study population, with n = 60 (63.8%), followed by patients with two-vessel disease (2VD) at n = 28 (29.8%) and three-vessel disease (3VD) at n = 6 (6.4%). Among patients with SVD, the left anterior descending artery (LAD) was the most commonly involved artery, affecting n = 48.9 (52.1%), followed by the left circumflex artery (LCX) in n = 67 (8.5%), and the right coronary artery (RCA) in n = 3 (3.2%) (Figure 3).

All patients in the study underwent complete revascularization. Prior to PCI, the average GLS was -16.72 ± 1.98. Twenty-four hours after PCI, the average GLS increased significantly to -19.89 ± 2.57 (p < 0.001). Six months post-PCI, GLS further improved significantly to -21.87 ± 1.70 (p < 0.001) (Table 3). This demonstrates myocardial recovery in our patient population, as evidenced by the significant improvement in GLS. The GLS values at different time points in the study were absolute.

**Table 3:** Overall GLS before and after intervention in the study population The data are represented as n (%) and mean ± SD. * p < 0.05 was considered statistically significant. GLS: global longitudinal strain; PCI: percutaneous coronary intervention

	N	Mean ± SD	Repeated measures ANOVA	
			F	p-value
Before PCI	94	-16.72 ± 1.98	228.90	<0.001*
24 hours post-PCI	94	-19.89 ± 2.57		
Six months post-PCI	94	-21.87 ± 1.70		

The increase in GLS was found to be statistically significant between pre-PCI and 24 hours post-PCI (p < 0.001), pre-PCI and six months post-PCI (p < 0.001), as well as between 24 hours post-PCI and six months post-PCI (p < 0.001) (Table 4).

**Table 4:** Comparison of overall GLS between different time intervals in the study population The data are presented as mean difference, SE, and CI. *p < 0.05 was considered statistically significant. GLS: global longitudinal strain; PCI: percutaneous coronary intervention

(I) Time	(J) Time	Mean difference (I-J)	SE	t-value	p-value	95% CI for difference	
						Lower bound	Upper bound
Before PCI	24 hours post-PCI	3.17	0.27	11.62	<0.001*	2.51	3.84
	Six months	5.15	0.23	21.99	<0.001*	4.58	5.72
24 hours post-PCI	Six months	1.98	0.22	9.06	<0.001*	1.45	2.51

**Figure 4:**
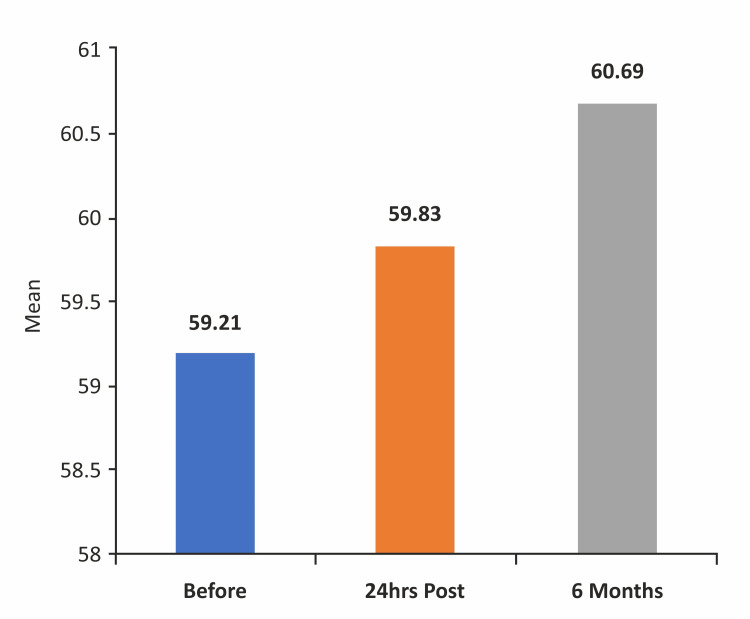
Comparison of GLS according to the number of coronary arteries stenosed before the intervention GLS: global longitudinal strain

Figure 4 illustrates a comparison of GLS according to the number of coronary arteries stenosed before the intervention.

Table 5 summarizes GLS before and after PCI according to the number of coronary arteries involved. Our study demonstrated a significant improvement in GLS in most patients across various subsets of the population, ranging from SVD to TVD, following stenting. However, these findings cannot be generalized due to the small size of our study population.

**Table 5:** GLS before and after PCI according to the number of coronary arteries involved * p < 0.05 was considered statistically significant. 2VD: double-vessel disease; 3VD: triple-vessel disease; GLS: global longitudinal strain; PCI: percutaneous coronary intervention; SVD: single-vessel disease

GLS	No. of arteries involved	N	Mean	SD	Minimum	Maximum	ANOVA	
							F	p-value
Before PCI	SVD	60	-17.41	1.75	-20.70	-12.80	18.47	<0.001*
	2VD	28	-15.91	1.61	-18.90	-12.20		
	3VD	6	-13.58	1.41	-15.00	-11.40		
24 hours post-PCI	SVD	60	-19.82	2.68	-26.80	-12.00	0.60	0.55 s(NS)
	2VD	28	-20.23	2.58	-26.70	-16.60		
	3VD	6	-19.03	0.80	-19.80	-17.60		
Six months post-PCI	SVD	60	-21.88	1.83	-26.00	-17.00	0.15	0.86 (NS)
	2VD	28	-21.78	1.56	-26.00	-19.40		
	3VD	6	-22.20	1.06	-24.20	-21.00		

Table 6 demonstrates the improvement in GLS at different time intervals in the study group, according to the number of coronary arteries involved. Most of our patients were symptom-free during follow-up. The two time points after PCI were considered to represent acute and chronic effects of revascularization, with the follow-up timings chosen based on analyses from similar studies. Many patients with TVD had greater derangement in GLS compared to those with SVD and therefore showed a more significant improvement after PCI. The normalization of GLS in TVD patients holds greater prognostic implications.

**Table 6:** Improvement in GLS between different time intervals in the study group according to the number of coronary arteries involved * p < 0.05 was considered statistically significant. 2VD: double-vessel disease; 3VD: triple-vessel disease; GLS: global longitudinal strain; PCI: percutaneous coronary intervention; SVD: single-vessel disease

No. of arteries involved	(I) Time	(J) Time	Mean difference (I-J)	SE	t-value	p-value	95% CI for difference	
							Lower bound	Upper bound
SVD	Before PCI	24 hours post-PCI	2.41	0.33	7.21	<0.001*	1.59	3.23
		Six months	4.47	0.28	16.04	<0.001*	3.78	5.15
	24 hours post-PCI	Six months	2.06	0.28	7.26	<0.001*	1.36	2.76
2VD	Before PCI	24 hours post-PCI	4.31	0.43	9.94	<0.001*	3.21	5.42
		Six months	5.86	0.33	17.96	<0.001*	5.03	6.7
	24 hours post-PCI	Six months	1.55	0.38	4.04	0.001*	0.57	2.53
3VD	Before PCI	24 hours post-PCI	5.45	0.59	9.19	0.001*	3.35	7.55
		Six months	8.62	0.56	15.39	<0.001*	6.64	10.6
	24 hours post-PCI	Six months	3.17	0.45	6.97	0.003*	1.56	4.77

In patients with SVD, baseline GLS was -17.41 ± 1.75, and there was a significant increase in GLS at 24 hours (-19.82 ± 2.68; p < 0.001) and six months post-PCI (-21.88 ± 1.83; p < 0.001). The improvement remained significant when comparing GLS between 24 hours and six months post-PCI (p < 0.001). In patients with DVD, baseline GLS was -15.91 ± 1.61, and there was a significant increase in GLS at 24 hours (-20.23 ± 2.58; p < 0.001) and six months post-PCI (-21.78 ± 1.56; p < 0.001). The improvement was also significant when comparing GLS between 24 hours and six months post-PCI (p < 0.001). In patients with TVD, baseline GLS was -13.58 ± 1.41, with a significant increase at 24 hours (-19.03 ± 0.80; p < 0.001) and six months post-PCI (-22.20 ± 1.06; p < 0.001). The improvement was significant when comparing GLS between 24 hours and six months post-PCI (p = 0.003).

In patients with LAD involvement, baseline GLS was deranged before PCI (-17.29 ± 1.75), and after PCI, GLS improved significantly at 24 hours (-19.89 ± 2.59; p < 0.001) and six months (-21.95 ± 1.81; p < 0.001) compared to baseline. The improvement at six months post-PCI was significantly greater compared to 24 hours (p < 0.001). In patients with LCX involvement, baseline GLS was not deranged before PCI (-18.09 ± 1.40), and after intervention, GLS improvement was not significant at 24 hours (-19.61 ± 3.64; p = 0.4), but was significant at six months (-21.63 ± 2.29; p < 0.002) compared to baseline. In patients with RCA involvement, baseline GLS was deranged before PCI (-17.60 ± 2.78), and GLS improvement was not significant at 24 hours (-19.27 ± 1.62; p = 0.16) or six months (-21.33 ± 1.15; p = 0.16) compared to baseline. The improvement at six months post-PCI was not significant compared to GLS at 24 hours (p = 0.21). The lack of statistical significance may be attributed to the small number of patients in this group.

In patients with SVD involving various arteries, the GLS values before intervention showed no statistical difference (p = 0.49). After PCI, although there was an improvement in GLS in all arteries, there was no statistical difference between the GLS values for different arteries at 24 hours (p = 0.91) and six months (p = 0.79).

Among 12 patients who underwent PCI to LAD+LCX, baseline GLS was deranged (-16.10 ± 1.44), and after revascularization, GLS improved significantly at 24 hours (-20.78 ± 2.79; p < 0.001) and six months (-22.03 ± 2.09; p < 0.001) compared to baseline. The improvement in GLS at six months was not significantly greater than at 24 hours (p = 0.29). Among 13 patients who underwent PCI to LAD+RCA, baseline GLS was deranged (-15.44 ± 1.77), and after revascularization, GLS improved significantly at 24 hours (-19.79 ± 2.61; p < 0.001) and six months (-21.52 ± 1.15; p < 0.001) compared to baseline. The improvement at six months was significantly greater than at 24 hours (p = 0.02). Only three patients (3.19%) underwent PCI to LCX+RCA, with baseline GLS deranged (-17.23 ± 0.74). After revascularization, the improvement in GLS was not significant at 24 hours (-19.93 ± 1.81; p = 0.30), but was significant at six months (-21.90 ± 0.26; p = 0.04) compared to baseline. In patients with 2VD involving various arteries, GLS values before intervention showed no statistical difference (p = 0.20). After PCI, although there was an improvement in GLS in all arteries, there was no statistical difference between the GLS values for different arterial combinations at 24 hours (p = 0.63) and six months (p = 0.73).

## Discussion

The present study was specifically designed to evaluate the utility of GLS measured by 2D-STE in detecting subclinical LV dysfunction in patients with stable CAD and to assess its improvement after revascularization. Our findings indicate that GLS at rest effectively predicted the severity of significant CAD in patients with stable angina who remained symptomatic despite OMT. Moreover, GLS provided additional diagnostic value over conventional echocardiography in identifying subtle LV dysfunction and determining CAD severity. This study also demonstrated significant early and late improvements in GLS in patients with CAD following revascularization. To our knowledge, this is the first study in the Indian population to assess the predictive value of GLS for detecting covert LV dysfunction in patients with significant CAD and its subsequent improvement after complete revascularization.

Our study provided evidence of subclinical LV dysfunction, as indicated by a baseline GLS of -16.72 ± 1.98, despite normal LVEF values of 59.21 ± 2.01. This discrepancy can be attributed to GLS measured using 2D-STE, a novel echocardiographic technique that evaluates global myocardial function by assessing the subendocardial longitudinal myocardial fibers. Abnormalities in these fibers become detectable long before any decrease in LVEF occurs [5]. LVEF, which reflects a combination of longitudinal and circumferential myofiber contraction, only begins to decline after substantial myocardial damage has already taken place, making GLS a more sensitive early marker of LV dysfunction than LVEF.

In the present study, we observed a progressive decline in GLS with increasing severity of CAD, as defined by the number of stenotic coronary vessels. This finding enhances our understanding of the mechanism of LV dysfunction in CAD patients, particularly as we excluded other concomitant causes of LV dysfunction. The risk of multivessel disease was found to increase as GLS decreased [12]. A severe reduction in GLS serves as an early indicator of overt LV dysfunction, which ultimately affects the quality of life of patients. Consequently, many centers are now using GLS to detect early myocardial damage caused by various pathologies.

Our findings are consistent with those of Radwan and Hussein [13], who reported a similar decrease in GLS with an increasing number of stenotic coronary arteries (GLS −18.65 ± 0.79, −15.13 ± 0.68, −12.25 ± 0.9, and −9.1 ± 1.94 for patients with nonsignificant CAD, SVD, 2VD, and 3VD, respectively; p < 0.0001). The study by Bala et al. [14] in 2018 also showed a comparable reduction in GLS with the increasing number of stenotic coronary arteries (GLS −18.27 ± 2.44 vs. −16.83 vs. −15.86 vs. −13.9 vs. −13.42 for patients with nonsignificant CAD, SVD, 2VD, 3VD, and left main (LM) disease, respectively; p < 0.001). Furthermore, our results align with those of Biering-Sørensen et al. [15], who found a similar pattern in GLS values (−18.8 ± 2.6 vs. 18.0 ± 2.4 vs. 16.7 ± 2.7 vs. −16.3 ± 2.3; p < 0.001) for patients with no significant CAD, SVD, 2VD, and 3VD, respectively. Additionally, the study by Choi et al. [16] reported comparable GLS values across different CAD categories, with patients having normal coronaries showing a mean GLS of −22 ± 1.5%, SVD or 2VD exhibiting −19.4 ± 2.4%, and triple-vessel or LM disease showing −18 ± 2.3%.

In all patients included in our study, GLS showed significant improvement after PCI. PCI is a widely used invasive treatment for CAD that alleviates symptoms, enhances LV function, and improves quality of life. In certain patient groups, PCI also reduces mortality rates [17]. Although PCI in patients with preserved LV function and OMT does not reduce cardiac death or MI, it helps decrease the need for additional procedures and lowers the risk of angina. The underlying pathophysiological benefit of revascularization lies in improving the function of viable myocardium [18].

Vanoverschelde et al. [19] established a correlation between the extent of structural changes and the rate of recovery, as determined through myocardial biopsies. They demonstrated that dysfunctional heart tissue in patients with significant stenosis can improve after revascularization. However, more sensitive techniques are required to assess the recovery of dysfunctional myocardium. The 2D-STE is a reliable method for monitoring early, subclinical LV changes. Steg et al. [20] highlighted that the recovery time of dysfunctional myocardium depends on the degree of cellular damage, which is influenced by factors such as the duration and severity of ischemia.

In our study, we observed a significant increase in GLS within 24 hours after PCI, compared to baseline values. Additionally, during the six-month follow-up, the measured GLS showed a further increase compared to the values recorded within 24 hours, indicating a trend toward the normalization of GLS.

The early improvement of GLS after revascularization suggests that restoring blood flow to the ischemic myocardium can enhance LV function in patients with CAD [21]. Our study demonstrated that recovery of LV function, detectable by GLS measurement, could be observed as early as 24 hours after revascularization. This finding aligns with several other studies that also showed early improvements in subtle LV dysfunction, as indicated by GLS. The early recovery of GLS is likely due to improved coronary flow post-PCI, reducing ischemia. In patients with DVD or 3VD, both early and late improvements were noted, suggesting that PCI offers beneficial effects.

Wang et al. [22] studied 40 patients and found GLS improved within 24 hours after PCI compared to baseline (-14.54 ± 2.06 vs. -13.25 ± 1.86; p < 0.001). Sodiqur et al. [21] also reported early recovery of LV function post-revascularization, with GLS showing significant improvement (-13.41 ± 4.94 vs. -12.41 ± 4.82; p < 0.0001). In a study by Hossain et al. [18], changes in echocardiographic parameters in 40 patients undergoing PCI revealed that GLS improved significantly within 48 hours after the procedure compared to baseline (15.0 ± 1.7 vs. 15.1 ± 1.7, p < 0.001). Our findings are also consistent with those of Yerajani et al. [23] (2021), who showed early recovery of LV function assessed through strain imaging.

However, our results were discordant with those of Magdy et al. [2], who studied 30 patients undergoing revascularization and found no significant early improvement in GLS within 24 hours (8.11 ± 3.3 vs. -8.95 ± 4.33; p=0.19). However, they did report significant late improvements at 3 months, with GLS improving in revascularized segments from baseline (-12.81 ± 3.27 vs. -8.11 ± 3.3; p < 0.01).

Late improvement of GLS after revascularization

In our study, significant late improvement in GLS was observed, which persisted at the six-month follow-up and was statistically greater compared to the improvement seen at 24 hours post-PCI. This finding aligns with several other studies. Chimura et al. [5] evaluated GLS before and nine months after PCI, demonstrating a significant improvement in GLS at the nine-month follow-up (-14.5 ± 4.1 vs. -12.4 ± 4.1; p < 0.01). Sikora-Frac et al. [17] conducted a study on 66 patients (CAD ± diabetes mellitus) and found significant late improvements in GLS at three months in both diabetic and non-diabetic patients following revascularization. Abd El Moneum et al. [24] (2019) compared GLS at six weeks post-revascularization in patients undergoing PCI or CABG for LAD disease, reporting a significant late improvement in the PCI group compared to the CABG group (-21.2 ± 6.5 vs. -21.2 ± 6.5; p < 0.001). Ha et al. [25] also reported a significant increase in peak systolic strain in ischemic segments six months after PCI in 22 patients with chronic stable angina.

In our study, patients with SVD showed significant improvement in GLS, both within 24 hours and at six months post-PCI. Among these patients, LAD was the most commonly involved artery (52.1%), followed by LCX (8.5%) and RCA (3.2%). This finding is consistent with studies by Sodiqur et al. [21] and Sikora-Frac et al. [17], where LAD involvement was predominant, and inconsistent with studies by Chimura et al. [5] and Wang et al. [22], which found RCA involvement to be more common.

We also observed that 29.8% (n = 28) of patients in our study had double-vessel disease (2VD), with LAD+RCA involvement being the most common (13.8%), followed by LAD+LCX (12.8%) and LCX+RCA (3.2%). This is comparable to Sodiqur et al. [21], who reported 27.5% of patients with 2VD, and Sikora-Frac et al. [17], who found 48% of patients with 2VD, with LAD+RCA > LAD+LCX > LCX+RCA. In patients with 2VD, GLS improvement from baseline was significant both at 24 hours and at six months post-PCI. The restoration of contractile function in the revascularized myocardium likely accounts for the improvement in LV function [26]. The degree of GLS improvement is influenced by the extent of myocardial jeopardy before revascularization.

A smaller subset of patients in our study (6.4%, n = 6) had 3VD. These patients showed significant GLS improvement from baseline both at 24 hours and at six months post-PCI. Patients with 3VD typically had higher SYNTAX scores, which reflect the anatomical complexity of CAD and may serve as an indirect marker of plaque burden [27]. A higher SYNTAX score suggests a greater plaque burden, which could result in more pronounced benefits from complete revascularization in this group of patients [17]. Similar improvements in GLS in patients with 3VD were reported by Hossain et al. [18] and Ryo et al. [28].

Limitations

This study highlights the utility of GLS as a bedside tool for detecting subtle LV dysfunction in patients with stable CAD who have normal LV function and no RWMAs. However, due to the small sample size, the true impact of this novel modality could not be fully assessed. Additionally, the GLS software used in echocardiographic machines is vendor-specific, and it remains unclear whether differences in software from various vendors would affect the comparisons across studies. Our study did not assess territorial longitudinal strain, and the potential impact of CABG on GLS in this population was not explored. The consistency of GLS measurements across different vendors is an important consideration for ensuring uniformity in clinical practice and research. We conducted our study using the GE Vivid 7 system, which is well-established for GLS measurement. Territorial longitudinal strain was not included in this study to avoid creating additional subgroups, but it is planned for future investigations. Similarly, the effect of CABG on GLS post-revascularization will be evaluated in future studies.

## Conclusions

Patients with stable CAD, normal LVEF, and no RWMAs on 2D echocardiography have been shown to exhibit subtle LV dysfunction when assessed by GLS, with improvement in GLS parameters following successful PCI. The derangement in GLS progressively increases from SVD to 3VD. This suggests that GLS derangement may be an early marker of LV systolic dysfunction in patients with ischemic heart disease, even when systolic function is preserved and RWMA is absent. Thus, 2D-STE-derived GLS could serve as a complementary bedside tool for assessing the impact of myocardial ischemia on LV systolic function. Early detection of subtle LV dysfunction through GLS may help prevent the progression to ischemic cardiomyopathy in patients with CAD. Notably, patients with 3VD show greater GLS derangement than those with SVD, indicating that identifying GLS abnormalities in patients with normal EF can be highly beneficial. Patients with more severe GLS derangement tend to have more complex CAD, emphasizing the need for early revascularization, potentially with multiple stents. In cases of high SYNTAX scores, CABG may play a more prominent role. GLS is a novel tool for identifying subclinical dysfunction caused by ischemic and non-ischemic factors, with the advantage of being easy to use and non-invasive. Future research should focus on larger, long-term studies to further validate the benefits of PCI on GLS, comparing it with other treatments such as CABG. Exploring the mechanisms behind GLS improvement and its prognostic value could enhance both understanding and clinical utility. These findings highlight GLS as a sensitive marker for subclinical LV dysfunction, supporting early intervention and personalized care strategies.
